# Determinants of people’s motivations to approach COVID-19 vaccination centers

**DOI:** 10.1038/s41598-023-30244-4

**Published:** 2023-03-31

**Authors:** Claudia Keser, Holger A. Rau

**Affiliations:** 1 University of Göttingen, Platz der Göttinger Sieben 3, 37073 Göttingen, Germany; 2 CIRANO, Montreal, Canada

**Keywords:** Human behaviour, Psychology, Health care, Disease prevention, Health care economics, Health policy, Public health

## Abstract

This paper presents the results of a survey exploring the determinants of vacinees’ confidence in COVID-19 vaccines and their motivations to become vaccinated. At the threatening rise of the highly infectious Omicron variant, in December 2021, we interviewed people in waiting lines of vaccination centers. Our results identify risk-averse and social-distancing-compliant people as showing high confidence in the vaccine, which motivates them to receive it for reasons of protecting themselves and others. By contrast, policy incentives, such as “3G/2G” restrictions, motivate risk-tolerant people who opted for vaccination to get access to public areas. Trusting people who regularly vote are little afraid of vaccines’ side effects. Our findings offer insights for policymakers in societies and firms that help to tailor policies promoting vaccination based on people’s economic preferences.

## Introduction

The COVID-19 pandemic that started in 2020 has caused a severe crisis for societies and the economy. To fight the dissemination of the virus, many states implemented policy measures, such as social-distancing rules, access restrictions, or lockdowns^1,2^. Compliance with these measures is an important prerequisite for a healthy society with functioning educational systems and labor markets. However, compliance as well as other behaviors related to the pandemic situation reflect a social dilemma, where negative externalities affect the whole society if citizens do not cooperate^3^. Examples relate to the break-down of the health-care system or the loss of human capital in the labor market, if too many people become seriously ill due to an unlimited spread of the virus^4–6^. In the short-run, missing workers lower output, causing gaps in supply chains with high economic costs for industries and the society. Potential long-term effects of a COVID-19 infection further increase the costs for the society.

In the battle against COVID-19, vaccines are a promising candidate to become a game changer. They do not only protect vaccinated persons against infection^7^ and a serious course of disease and hospitalization^8^, but also provide positive externalities on the society in that a very high vaccination rate may curb the spread of the virus. However, many countries face the problem that the vaccination rate is too low. In August 2022, it is 67% in the USA, 75% in the UK, and 76% in Germany according to “Our World in Data”^9^. An important reason for an insufficient vaccination rate in a country is a low degree of acceptance of COVID-19 vaccines among its citizens.

In the summer of 2021 and in the winter of 2021/2022, many German federal governments introduced “3G” and ”2G” policies offering access to public areas (e.g., bars, restaurants and fitness centers) only to those who are vaccinated (“**G**eimpft”) or recovered from a previous COVID-19 infection (“**G**enesen”) in the case of “2G” or alternatively  tested negative (“**G**etestet”) in the case of “3G”. Furthermore, boostered people have been offered shorter quarantine times and an exemption of the need for a negative COVID-19 test to access locations under a “2G+” rule. From a behavioral economics perspective, such policies may be interpreted as vaccination incentives. Experimental evidence shows that financial incentives may increase vaccination rates in real^10^ and in hypothetical scenarios^11^. However, little is known on the success of restriction policies and the determinants of people’s vaccination motivations. Therefore, a better understanding is needed, which helps to design policies that increase vaccination motivations.

In this paper, we fill this research gap with a survey study in Göttingen (Lower Saxony, Germany) that analyzes the determinants of vaccination motivations of people who want to get vaccinated. Precisely, we surveyed people who approached two mobile vaccination centers in Göttingen, which offered on a first-come-first-serve basis and without preregistration COVID-19 vaccination on December 3, 10, and 17 in the year 2021. We employed two research assistants that conducted the surveys with people just prior to passing by a registration desk before they could get the vaccine.

To take into account the impact of the recent policy changes on subjects’ motivation, we conducted the study shortly after the introduction of the 2G+ rule (December 1, 2021) and around the introduction of the exemption of boostered people of the need for a negative test under the 2G+ rule (December 4, 2021) in Lower Saxony.

A growing literature applies survey studies on vaccine acceptance and finds that it is curbed by increased distrust in politics^12^, science^13^, citizens’ beliefs in conspiracy theories^14–17^, and a lack of information about COVID vaccines^18^. Moreover, it has been observed that women have a lower vaccine acceptance^19^ and are more afraid of side effects than men^20^, and that people show a lower acceptance of vaccines, when hypothetically assessing the scenario of compulsory vaccination^21^. Although, this literature adds important insights on the determinants of vaccination, a shortcoming is that all these studies rely on hypothetical answers whether people intend to get vaccinated, or it is unknown whether they are really vaccinated.

It is a strength of our study that, when surveying people in waiting lines of vaccination centers shortly before they receive the vaccine, we know that they most likely received the vaccine. Otherwise, had we just asked people about their vaccination intention, their answers might have been subject to a desirability bias. While we get rid of this bias in our study, we face a selection bias in that our sample excludes vaccination refusers. Such participation bias is a common problem, which however may also occur in many large survey studies on COVID-19 uptake^22^.

Following two studies showing that economic preferences predict people’s behavior in the pandemic^3,23^, we elicit subjects’ risk tolerance, trust, patience, altruism, social-distancing behavior, and their willingness to go voting. The goal of our study is to test whether these preferences and behaviors determine people’s confidence in the vaccine and their motivation to uptake the vaccine. Our participants rate their vaccination motivations in categories, such as protection issues, getting access to restricted areas, and peer pressure. Analyzing the role of preferences for subjects’ vaccination motives allows us to elaborate, what kind of people were attracted by incentivizing policies.

Our results show that compliant, risk-averse people, who feel well-informed about the COVID-19 vaccination, believe in vaccines’ efficacy and want them for reasons of protection and fear of Omicron. We find evidence of a gender effect in that significantly more women than men are motivated by protection and fear issues. By contrast, the incentivizing policies apparently attract risk-tolerant and less altruistic people, who state that they decided to get vaccinated because of the motive of getting access to restricted public areas. Moreover, risk-tolerant subjects are also motivated by getting the vaccination because of reasons of peer pressure. Finally, we find that trusting people are less afraid of side effects, which is in line with the literature^21^. Our correlation results can be used by policymakers and firms for the “prediction problem”^24^ when designing tailor-made policies and institutions. Precisely, they may apply policies that grant access to restricted areas (or payment schemes) conditional on being vaccinated in regions or firms characterized by a high concentration of risk-tolerant people.

## Methods

### Questionnaire design

To collect the data of this study we surveyed people who approached two mobile vaccination centers in Göttingen, which offered on a first-come-first-serve basis and without preregistration COVID-19 vaccination on December 3, 10, and 17 in the year 2021. We employed two research assistants that interviewed people, who were willing to get the vaccine and therefore approached the mobile vaccination center. They had to pass by a registration desk (medical interview) before they could get the vaccine. When recruiting the participants, the research assistants asked all vaccinees that passed by the registration desk, whether they wanted to participate in a short questionnaire on vaccination motives that would not last more than ten minutes. In this respect, informed consent was obtained from all the participants. Our sample size is not based on sample calculations since the questionnaire was conducted in short-term nature on three days due to the dynamics of changes of COVID-19 restrictions at this time.

### Study participants, ethical consideration

Informed consent was obtained from all participants. In accordance with the Declaration of Helsinki, all participants were shown a consent form that guaranteed the anonymity of the data generated during the survey. All participants agreed with its terms before taking part in our survey. For the GLOBE Lab of the University of Göttingen, there is a general IRB approval, which was approved by the University of Göttingen. Our study procedure is in line with the ethical guidelines stated in this approval.

Participants were also told that they would receive a chocolate bar as a reward for participation. Our survey study is divided into three blocks, the elicitation of participants’ (1) preferences and voter turnout, (2) perceptions of the COVID-19 vaccination, and (3) motivations to become vaccinated. We apply packages of questions to address measurement error. The research assistants collected most of the data (84%) at the mobile vaccination center located in the lecture hall building on the central campus, whereas the remaining data were collected at the mobile vaccination center in the rooms of a former university restaurant in the city center. In total, we collected data of 172 participants (54% female), aged 33.31 (sd: 16.57) on average, and 88% having an academic background (current or formerly completed studies). The mean age (main campus: 33.44; city center: 33.04) and the fraction of participants who indicated that they study or formerly completed their studies (main campus: 89%; city center: 85%) are similar in both locations. Due to missing information, where participants did not give an answer, or due to the indication of non-binary gender, the main data are based on 156 observations. In our regression analyses, where we apply additional control variables including subjects’ COVID-19 experiences, we have data of 154 participants, since two participants did not have an answer to this question. Since we approached our participants at the vaccination centers, it is quite likely that they intended to become vaccinated. This allows us to derive important conclusions on vaccinated people’s confidence in COVID-19 vaccines and the underlying motivations of their vaccination decisions. This is a strength of our study as compared to standard survey studies, where researchers do not know, whether people claiming to be vaccinated actually tell the truth. Those studies might suffer from a social desirability bias regarding participants’ statements on their vaccination status. A shortcoming of our study is that we have to rely on a selected sample, i.e., people who approached the vaccination center. Note also that the vaccines were rationed on each day of our data collection, such that participants did not know for sure, whether they would manage to receive the vaccine, when we interviewed them in the queue.

### Question types

The first block (1) of the questionnaire applies a package of verbal questions on economic preferences, similar to those investigated in a related survey study on compliance behavior^3^ and based on papers regarding the role of (non-)incentivized measurement of economic preferences^25,26^. Since vaccination decisions involve uncertainty related to the vaccines’ efficacy and potential side effects, we elicit subjects’ risk tolerance and trust in other people on 11-point rating scales (0 = the lowest degree; 10 = the highest degree). We measure time preferences, by asking participants about the immediate compensation in Euros they would request to forego a payment of €1000 in six months. We also ask them about their required compensation in six months to give up a payment of €1000 in twelve months. We use the mean of the two measures to account for patience, assuming that more (less) patient subjects claim a higher (lower) amount. Time preferences have shown to be of importance in the context of compliance with health regulations^3^. We pose a verbal question on charitable giving to proxy altruistic preferences: how much money, out of ten 1-Euro coins in your wallet, would you donate, when walking along the street passing by a charity representative asking for a donation. We use the donation amount as a proxy for altruism, which is in keeping with experimental dictator-games with charities as recipients^27^. We expect altruism, to be of relevance in the context of vaccination decisions that may also protect others^28^. To account for subjects’ political participation, we ask participants on their assessment of the importance to vote on an 11-point rating scale (0 = totally unimportant; 10 = very important).

The second block (2) consists of contextual questions regarding participants’ confidence in the COVID-19 vaccination. Based on 11-point rating scales (0 = not at all; 10 = very good) we measure participants’ assessments of the vaccination’s protection against COVID-19 and their perception of how well they feel informed about the vaccines. Furthermore, participants state their fear of vaccine side effects(0 = not afraid; 10 = very afraid). Finally, we ask them whether today’s vaccination would be their booster vaccination. For those who had previously received two doses of the Biontech/Pfizer, Moderna or AstraZeneca, or a first dose of the Johnson & Johnson COVID-19 vaccines, we count the next vaccination as booster.

The third block (3) consists of contextual questions on participants’ motivations to receive the COVID-19 vaccination and on their experiences and behavior in the pandemic. First, participants answer questions on their vaccination motives on 11-point rating scales (0 = does not apply; 10 = does perfectly apply): (i) Self protection against COVID-19; (ii) protection of fellow people; (iii) fear of the new Omicron variant from South Africa; (iv) getting access to public areas such as retail, restaurants and fitness centers with “3G/2G” requirements, which were in place in Germany during the time of our study and which implied that only those, who were either vaccinated or had recovered from COVID-19 (“2G” rule) or only those, who either were vaccinated or had recovered from COVID-19 or can prove a negative recent test result (“2G” rule), get access. Then, participants state the extent of their personal illness experience with COVID-19 (0 = not at all ill; 10 = hospital). Next, they indicated to what extent they keep distance to fellow people in public during times of high incidence values (0 = not at all; 10 = completely). We use this measure as a proxy for compliance, assuming that higher scores reflect increased compliance. Moreover, we ask subjects whether they regularly have contact to people who are at risk. Finally, we collect their demographics (age, gender, nationality, whether they study or have studied).

### Data analysis and construct validity

We standardize all variables, except the dummy variables. Our main analyses focus on regressions that investigate the determinants of two different dimensions, (i) confidence in the vaccine and (ii) motivations to get vaccinated. The first dimension, confidence in the vaccine, measures an important basis for people’s acceptance of COVID-19 vaccines. In this respect, we consider (and run regressions on) two contrary aspects, believed efficacy of the vaccine and the fear of potential side effects. The second dimension, motivations to get vaccinated, aims to dig deeper into subjects’ vaccination motives by distinguishing between precautionary measures vs. reasons of increased personal freedom and social status. In this respect, we run regressions that study the determinants of subjects’ motivations to receive the vaccine for reasons of protection and fear, for getting access to restricted areas, and for reasons of peer pressure.

Since the protection motivation is multidimensional and in order to address measurement error, we ask several questions to account for it. Based on the answers to these questions, we compute a protection & fear index as an outcome variable. Following related studies^3,29^, we take the arithmetic mean of various variables that we think are theoretically connected. These include participants’ answers regarding their motivations based on: “self-protection,” “protection of others,” and “their fear of Omicron.” We believe that the two reasons of protection are closely interrelated, since both motivations depend on the risk of getting infected and spreading the virus. The same should be true for subjects’ fear of the Omicron variant. Cronbach’s alpha shows sufficient reliability of the protection index (0.602).

In all of our regression models, we use a set of variables on economic preferences, compliance, and political participation. We conduct a principal component analysis (pca) to reduce the number of variables and to identify specific types of relevant combinations^3,30^. Motivated by previous findings that highlight the importance of economic preferences and social responsibility to predict behavior during the pandemic^3^, we include our data of economic preferences (risk tolerance, time preferences, altruism, trust in others) and social responsibility (voting behavior, social distance and compliance) in the pca. Moreover, we include subjects’ perception of how well they feel informed about the vaccination, as there is evidence that information on the COVID-19 vaccination affects subjects’ self-efficacy to become vaccinated^31^.

In the pca, factors are extracted based on eigenvalues above one, which is in line with Kaiser’s rule. A factor loading of greater than 0.50 is used to identify items. We identify four components with eigenvalues exceeding one. Afterwards, a varimax rotation is applied. As a result, in component one, two items load positively and very strongly, trust others (0.71) and voter turnout (0.56). The component can be interpreted as reflecting the characteristics of a trusting person who feels obliged to vote. Therefore, we call the first principal component (pc) “PC1: Trust others & Political participation.” In component two, two items load positively and very strongly, complying with social distance (0.81) and feeling well-informed on the vaccine (0.56). Thus, the pc reflects the characteristics of a compliant person who feels well-informed. Therefore, we label it “PC2: Compliant & Feels informed.” In component three, results show that donations to the charity loads positively and very strongly (0.75). Whereas, mean patience loads negatively (− 0.60). Thus, we call the pc: “PC3: Altruistic & Impatient.” Finally, in component four, only risk tolerance loads very strongly (0.81). Thus, we label it “PC4: Risk tolerant”.

## Behavioral predictions

In this section, we derive behavioral predictions on the determinants of (i) people’s confidence in COVID-19 vaccines and (ii) their motivations to get vaccinated.

With respect to our first dimension, confidence in vaccines, we focus on previous survey findings on people’s COVID-19 vaccine acceptance. Trusting people show a higher acceptance of COVID-19 vaccines^21,32^, which should be reflected in increased confidence about the efficacy of the vaccine. Moreover, we expect that people who want to become vaccinated are more convinced that the vaccine protects them against COVID-19, when they feel well-informed about the vaccine.

Evidence on individual risk preferences suggests a negative relationship between risk tolerance and willingness to take health risks^26,33^. Thus, we expect that risk-tolerant people are less convinced about the efficacy of vaccines and show less fear of side effects.

*Behavioral prediction 1: confidence in the vaccines*

(a) Trust is positively related with confidence in vaccine efficacy and negatively related with the fear of side effects.

(b) Feeling well-informed is positively related with confidence in vaccines’ efficacy.

(c) Risk tolerance is negatively related with confidence in vaccine efficacy.

With respect to the determinants of people’s motivation to become vaccinated, we expect that people who follow the rules are convinced of the meaningfulness of government recommended COVID-19 vaccines as a means to protect against the virus. We expect that altruistic people vaccinate against COVID-19 to protect others, given the evidence that influenza vaccines are commonly used to protect others^28^. Moreover, risk-averse people are known to uptake vaccines for protection issues^34^. By contrast, less altruistic and risk-tolerant people should be attracted by reasons of increased personal freedom. That is, they may be the people who are attracted by the incentives to receive the vaccine for accessing restricted areas. Moreover, we expect that risk-tolerant people are more likely to receive the vaccine for reasons of peer pressure. Since they are not afraid of the virus, it is possible that they vaccinate because of social status.

*Behavioral prediction 2: motivations to get vaccinated*

(a) Compliance, altruism and risk aversion are positively related with the motivation to receive the vaccine for protection issues.

(b) Altruism is negatively, and risk aversion is positively related with the motivation to get vaccinated for accessing restricted areas.

(c) Risk tolerance is positively related with the motivation to become vaccinated because of peer pressure.

## Results

Before we turn to our main results on participants’ vaccination motives, we analyze the determinants of people’s confidence in COVID-19 vaccines.

### Confidence in COVID-19 Vaccines

We analyze in this section the determinants of people’s confidence in the vaccine’s efficacy and their fear of side effects. Overall, we find that 90% of people who approached the vaccination centers expected their booster COVID-19 vaccination, which corresponds to their third vaccination. Whereas, 7% (3%) of the people did not receive any (received one) COVID-19 vaccination before. The findings are in line with the German phenomenon of that time, that only a small percentage of people approached vaccination centers to receive their initial vaccination. Unfortunately, due to the small percentage of initial vaccinees, we cannot investigate people’s motivations for late initial vaccinations.

**Figure 1:**
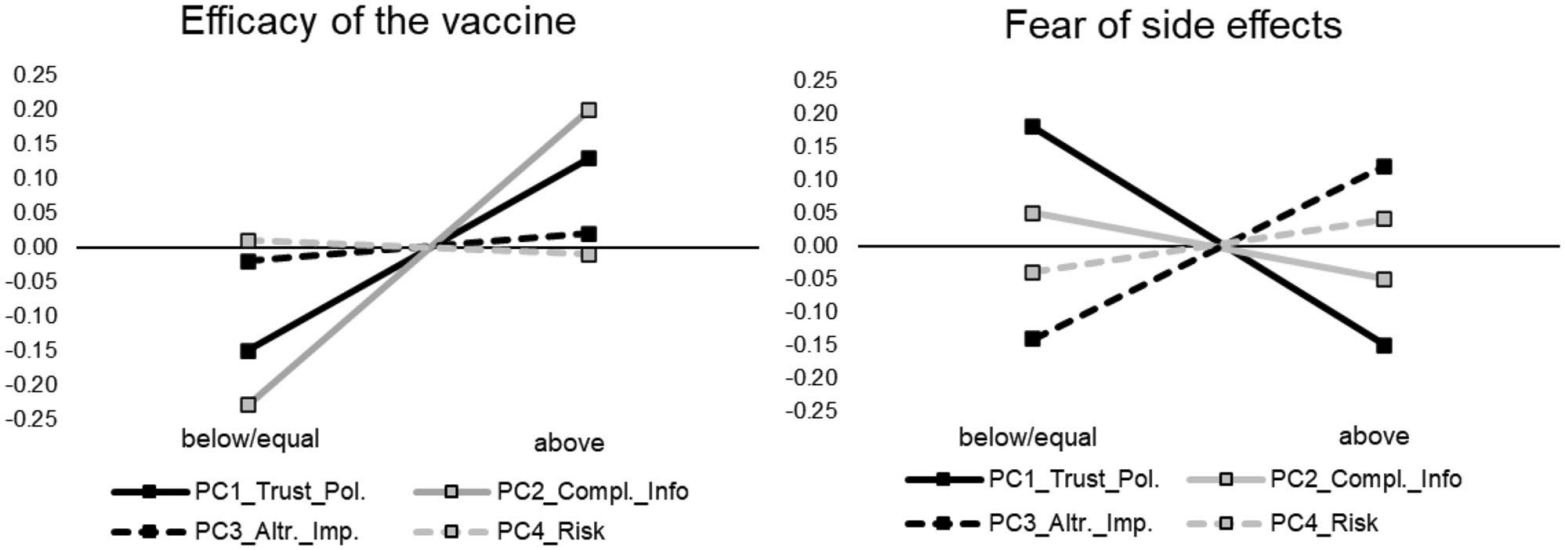
The determinants of people’s confidence in the vaccine and of their fear of side effects.

We start our analysis with an overview on the impact of our principal components (PCs). Figure 1 displays an overview on the relation of our PCs on the “efficacy of the vaccine” (left panel) and their “fear of side effects” (right panel). We focus on median splits of the PCs. In each panel, PC1 (PC2) is represented by the black (gray) solid line. Whereas, PC3 (PC4) is represented by the black (gray) dashed line.

The most conspicuous finding in the left panel is that people with an above-median level of PC2 have a higher belief (0.20) of the vaccine’s efficacy than people with a below/equal median PC2. Thus, compliance and being well-informed about the vaccine, seem to be positively correlated with confidence in the efficacy of the vaccine. The diagram also suggests that a higher PC1 has a similar, though less pronounced, effect (− 0.15 vs. 0.13). Turning to people’s fear of side effects, the most obvious effect that we observe in the right panel is that a higher PC1 is related with less fear of side effects. Thus, above-median trusting people who vote are less afraid of sides effects (− 0.15) than those with a below/equal-median value of PC1 (0.18). In the next step, we make use of parametric-regression analyses to test for statistical significance.

### Regression analyses

Table 1 presents OLS regressions on subjects’ confidence in vaccines, focussing again on the two aspects, subjects’ perception of the efficacy of the vaccine (models (1)–(2)) and their fear of possible side effects (models (3)–(4)).

**Table 1:** OLS regressions on subjects’ trust in the COVID-19 vaccines.

	Efficacy of the vaccine		Fear of side effects	
	(1)	(2)	(3)	(4)
PC1: Trust others & political participation	0.132*	0.064	− 0.243***	− 0.149**
	(0.069)	(0.071)	(0.067)	(0.069)
PC2: Compliant & feels informed	0.212***	0.239***	− 0.044	− 0.056
	(0.071)	(0.071)	(0.069)	(0.069)
PC3: Altruistic & impatient	− 0.017	0.037	0.096	0.034
	(0.072)	(0.094)	(0.070)	(0.092)
PC4: Risk tolerant	− 0.137*	− 0.155**	− 0.002	− 0.044
	(0.072)	(0.078)	(0.070)	(0.076)
Female	− 0.130	− 0.173	0.254*	0.231
	(0.156)	(0.152)	(0.151)	(0.148)
Age		− 0.242***		− 0.131
		(0.085)		(0.083)
Current/former student		− 0.294		− 0.573*
		(0.312)		(0.303)
German		0.228		0.145
		(0.346)		(0.337)
Contact with people at risk		0.002		0.148
		(0.160)		(0.156)
COVID-19 experience		− 0.118		− 0.015
		(0.072)		(0.071)
Receive booster vaccination		1.040***		− 1.053***
		(0.367)		(0.357)
Constant	0.061	− 1.019*	− 0.168	1.370**
	(0.115)	(0.562)	(0.112)	(0.548)
Controls for location and wave	no	yes	no	yes
obs.	156	154	156	154
$R^{2}$	0.112	0.242	0.107	0.233
Standard errors in parentheses				
***$p<$0.01, **$p<$0.05, *$p<$0.1				

Our basic models contain in regressions (1) and (3) the estimated principal components (PCs). PC1: Trust others & Political participation is our first PC, where trust and voter turnout load high and positive. PC2: Compliant & Feels informed is our second PC, with high positive loading for subjects who take great care about social distancing and who feel well-informed about the vaccine. PC3: Altruistic & Impatient is our third PC with a high positive loading for subjects who donate much and a negative load of patience. PC4: Risk tolerant is our fourth PC with a high positive loading for risk-tolerant subjects. In our regressions, we also control for gender effects with female, a dummy that is positive for women. Models (2) and (4) add control variables. Age is subjects’ age in years, current/former student is a dummy, which is positive for students (or those who formerly have completed studies). German is a dummy that is positive for German participants. We also add a dummy variable (contact with people at risk) that controls whether our participants commonly meet people endangered by the Coronavirus. We also control for the impact of participants’ reported level of the experience with the virus (COVID-19 experience). Moreover, we include a dummy that is positive for those participants who attempt to receive their booster vaccination. Finally, we include a variable wave to account for possible time dynamics during the pandemic. The variable is one when the data collection was on December 3, it is two when it was on December 10, and it is three when it was on December 17. Moreover, we add a location dummy, which is one (zero) when the data were collected at the campus (city center). All regressions report standard errors in parentheses.

Models (1) and (2) highlight that compliant and informed people believe in the effectiveness of the vaccine. That is, the coefficient of PC2 is significant and positive, i.e., a one standard deviation (sd) increase leads to a 0.239 sd increase in subjects’ perceived efficacy of the vaccines. Moreover, PC4 is significantly negative, i.e., risk-averse (or, less risk tolerant) subjects believe that the efficacy of the vaccines is higher. We also find that PC1 is positive and weakly significant. However, Model (2) confirms that this effect is not robust when adding controls. By contrast, the model confirms the effects of PC2 and PC4. Model (2) reveals a highly significant negative relation with age, i.e., younger people are more convinced about the efficacy of the vaccine. Finally, we find a highly significant positive relation for people who plan to receive a booster vaccination. That is, people who frequently received the vaccine believe that it works. The findings confirm the pattern of Fig. 1.

*Result 1* Compliant and risk-averse people who want to get vaccinated and feel well-informed about the vaccine are convinced about its efficacy. Younger people show higher confidence in the vaccine.

Turning to people’s fear of side effects, we find a strong negative relation for PC1. That is, trusting people who show a high political participation are less afraid of side effects. Model (3) shows that this effect is of slightly higher magnitude than the effect of PC2 in Model (1). It turns out that a one sd increase in PC1 leads to a 0.243 sd decrease in subjects’ fear of side effects. Model (4) shows that the effect is robust when adding control variables, although the effect becomes weaker in this case. Model (3) documents that women tend to be more afraid of side effects than men, which is in keeping with the literature on the perception of vaccines^20^. However, the effect is not robust in Model (4). Model (4) shows that younger people and current or former students tend to be less afraid of side effects. In line with that, we also find that the campus dummy is negatively significant. Furthermore, results show an intuitive highly significant negative relation of the dummy, which controls whether subjects come to receive a booster vaccination and their fear of side effects. Put differently, people who plan to receive the booster vaccine show lower levels of fear of side effects. Again, the results are in line with the pattern of Fig. 1.

*Result 2* Trust and political participation of people ready to get vaccinated are associated with less fear of side effects.

In summary, we find that trust is negatively associated with people’s fear of side effects, but its positive association with confidence in vaccine efficacy is not robust to the inclusion of control variables. Moreover, we find strong support that the feeling of being well-informed is positively associated with people’s confidence in the vaccine’s efficacy, which also holds for risk-averse people. Thus, our results are in line with Behavioral Predictions 1a–1c.

### Motivations to become vaccinated

**Figure 2:**
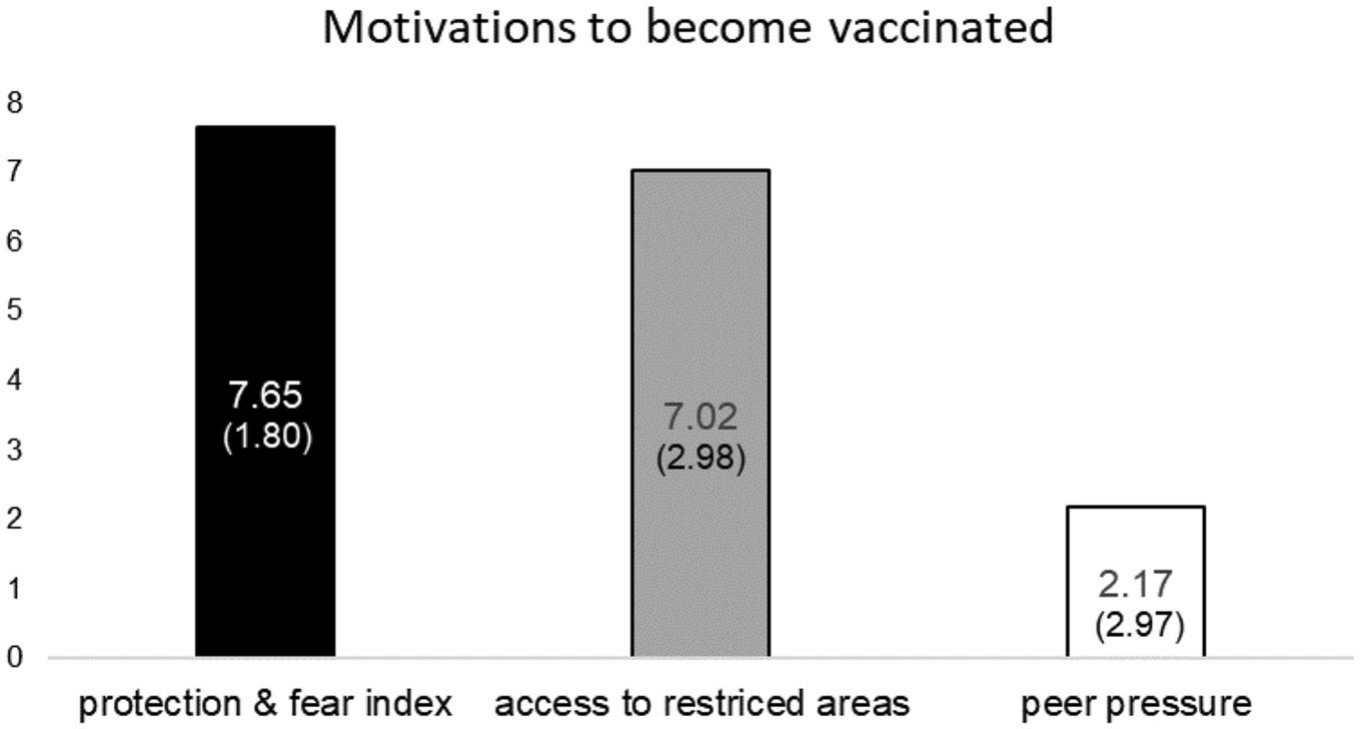
Participants’ stated motivations to become vaccinated.

Next, we report our findings on the determinants of contrasting motivations of receiving the vaccine. Note that these reasons are mainly related to subjects’ motivations to receive a booster, since 90% of our subjects received a booster. We compare the determinants of precautionary measures vs. motives of increasing the personal freedom vs. motives of keeping social status. Before that, we present an overview of the means of participants’ answers regarding the vaccination motives. This is displayed by Fig. 2. The black bar presents the mean of the protection & fear index. Whereas, the gray (white) bar presents the mean score of receiving the vaccine because of reasons of access to restricted areas (peer pressure).

Results show that the scores of the “protection & fear index” and “access to restricted areas” are significantly higher than “peer pressure” (Wilcoxon sign-rank tests, p < 0.001). Whereas, no significant difference occurs between “protection & fear index” and “access to restricted areas” (Wilcoxon sign-rank test, p = 0.121).

Our main results on the determinants of vaccination motives are summarized in Table 2. It presents OLS regressions on the three motivations to receive a vaccine, which we have discussed above. The first motivation is directly related to the Coronavirus in that it focuses on protection issues and subjects’ fear of Omicron. It is captured by our index protection & fear (models (1)–(2)). The second motivation analyzed in models (3)–(4) concerns the effect of receiving the vaccine to get access to restricted areas, such as the retail sector or restaurants and bars. Finally, we consider peer pressure as a motivational factor to maintain one’s social status by getting vaccinated (models (5)–(6)). In all these models, we correspondingly apply the same independent variables and controls as in Table 1.

**Table 2:** OLS regressions on subjects’ motivations to become vaccinated.

	Protetction & fear index		Access to restricted areas		Peer pressure	
	(1)	(2)	(3)	(4)	(5)	(6)
PC1: Trust others & political participation	0.175**	0.083	0.016	0.031	− 0.037	0.029
	(0.068)	(0.067)	(0.071)	(0.076)	(0.070)	(0.073)
PC2: Compliant & feels informed	0.249***	0.230***	− 0.126*	− 0.104	− 0.005	− 0.012
	(0.070)	(0.066)	(0.074)	(0.075)	(0.072)	(0.072)
PC3: Altruistic & impatient	0.034	0.149*	− 0.229**	− 0.227**	− 0.002	− 0.062
	(0.070)	(0.088)	(0.091)	(0.100)	(0.072)	(0.096)
PC4: Risk tolerant	− 0.061	− 0.160**	0.159*	0.157*	0.201***	0.150*
	(0.070)	(0.073)	(0.082)	(0.083)	(0.072)	(0.081)
Female	0.471***	0.386***	0.087	0.082	0.216	0.222
	(0.152)	(0.143)	(0.163)	(0.162)	(0.158)	(0.157)
Age		0.020		− 0.034		0.008
		(0.080)		(0.090)		(0.088)
Current/former student		− 0.496*		− 0.242		− 0.706**
		(0.290)		(0.331)		(0.319)
German		0.209		− 0.329		− 0.311
		(0.322)		(0.369)		(0.354)
Contact with people at risk		0.092		− 0.286*		− 0.268
		(0.151)		(0.171)		(0.165)
COVID-19 experience		0.074		− 0.175**		0.084
		(0.067)		(0.077)		(0.075)
Receive booster vaccination		1.674***		0.103		− 0.515
		(0.341)		(0.390)		(0.376)
Constant	− 0.290**	− 1.608***	− 0.028	0.487	− 0.129	1.797***
	(0.113)	(0.524)	(0.119)	(0.598)	(0.117)	(0.576)
Controls for location and wave	no	yes	no	yes	no	yes
obs.	154	152	154	153	154	152
$R^{2}$	0.183	0.350	0.069	0.143	0.062	0.167
Standard errors in parentheses						
***$p<$0.01, **$p<$0.05, *$p<$0.1						

In models (1)–(2), we find a strong effect for PC2, which is positive and highly significant. Thus, compliant subjects who feel well-informed want to receive the vaccine to protect themselves and others. Another reason is that they fear Omicron. Precisely, model (2) shows that a one sd increase in PC2 leads to a 0.230 sd increase in subjects’ motivation to receive the vaccine for reasons of protection and fear. Model (1) indicates a significantly positive effect of PC1. However, this effect disappears in Model (2), when applying control variables. Model (2) reveals a significantly negative effect of risk tolerance, i.e., risk-averse people are motivated to protect others. Precisely, a one sd increase in risk tolerance leads to a 0.160 sd decrease in subjects’ motivation to receive the vaccine for reasons of protection & fear. Finally, Model (2) shows a significantly positive relation between altruistic people and the motive to get vaccinated for protecting others. Taken together, we find support for Behavioral Prediction 2a. Moreover, models (1)–(2) highlight a gender effect. That is, the coefficient of female is positive and highly significant. Thus, it is particularly the women who are afraid of Omicron and who receive the vaccine for reasons of protection. Finally, we find that the dummy controlling for the booster vaccination is positive and highly significant. Thus, people, who want to receive a booster vaccine, do this particularly for reasons of protection and fear of Omicron. A closer look at disaggregated analyses of the components of the index reveals that the gender effect is particularly driven by women’s pronounced motivation to protect others (see Table S1 in the Supplementary Information). In addition, we find that women are more afraid of Omicron than men.

*Result 3a* Compliant and risk-averse people who feel well-informed and are ready to get the vaccine want it for reasons of protection and fear. It is particularly the women, who want the vaccination for protective issues.

A main finding in models (3)–(4) is that the coefficient of PC3 is significantly negative. Pearson’s correlation coefficients show that this relation is entirely driven by altruism (Pearson’s correlation coefficients, altruism: $\rho$ = − 0.171, p = 0.027; patience: $\rho$ = − 0.021, p = 0.793). Thus, less altruistic people want the vaccine for motives of getting access to restricted areas. The effect is of the same size as the effect of PC2 in model (2). That is, a one sd increase in PC3 leads to a 0.227 sd decrease in the access motivation. Moreover, we find a weakly significant effect for PC4. Thus, it is the risk tolerant people who tend to be motivated by reasons of getting access to restricted areas. The results find support for Behavioral Prediction 2b. Moreover, model (4) reveals that a motivation to become vaccinated because of reasons to get access is more likely when people have no contact to people at risk. Finally, we find a significantly negative effect of COVID-19 experience. Thus, people who had only minor personal experience with COVID-19 want to get the vaccine for motives of personal freedom.

*Result 3b* Less altruistic and more risk tolerant people that want to get vaccinated want the vaccine for reasons of getting access to restricted areas. People that have contact to people at risk and people who had strong personal experiences with COVID-19 are not motivated by this reason.

Models (5)–(6) highlight significantly positive coefficients of PC4: Risk tolerant. The effect becomes less pronounced, though, when adding controls. Thus, peer-pressure motives matter for risk-tolerant people. In model (6), we find that a one sd of PC4 leads to a 0.150 sd increase in peer-pressure motives. The finding is in line with Behavioral Prediction 2c. Moreover, model (6) shows that current or former students are less motivated by peer pressure.

*Result 3c* Peer-pressure motives matter for risk tolerant subjects, whereas they are less important for current or former students.

## Discussion

In times of the pandemic, a key goal is the avoidance of high infection rates, in order to prevent congestion in hospitals and the lack of human capital in labor markets. A report based on data of 2020^4^ suggests that a one-per-thousand increase in infections caused a two-to-three-percent drop in local employment in Korea. Thus, vaccines fighting the spread of the virus are a central mean to guarantee economic growth during the COVID-19 pandemic. In our paper, we studied individual motives to get vaccinated and their determinants by applying economic preferences, individual compliance and voting behavior. An advantage of our approach is that we interviewed people in waiting lines of mobile vaccination centers who ultimately decided to get vaccinated. This attenuates problems of social desirability bias.

We find that economic preferences and compliance are predictors of citizens’ confidence in the vaccine and their motivations to receive it. We identify risk-averse, compliant people, as showing high confidence in the vaccine. Moreover, their risk attitude explains their motivation to receive the COVID-19 vaccine to protect themselves and others against the virus and newer variants, such as Omicron. The same is true for female participants, who are particularly motivated by protection issues. By contrast, risk-tolerant and less altruistic people are attracted by policies granting access to restricted areas, or they receive the vaccine because of status effects. Finally, our results reveal that trusting people who participate in voter turnout are not affected by fear of vaccine side effects.

A limitation of our study is that we focus on a rather small sample of people approaching a vaccination center to receive the vaccine that mainly encompasses current or former students. Therefore, the sample excludes vaccination refusers, which complicates the generalization of the results. As a consequence, policy implications only hold for people who are in principle ready to become vaccinated. To derive more tailored policy implications, more data are needed.

Nevertheless, our results of the pca add interesting first insights into the correlations of the preferences of people that definitely want the vaccine and their motivations to do so. To increase the generalizability, more evidence, in particular, data on vaccination refusers is needed. Although, students might not be representative for the whole population, they represent an important age cohort, which was documented as least compliant with Corona regulations^35–37^. Thus, we focus on an age cohort that is of importance for vaccination campaigns. The behavior of students is important for firms, as they represent an age group that is pretty close to enter the labor market. Student subject pools have the advantage that they are homogeneous and therefore less prone to measurement error. It was shown in a large study that their behavior correlates with a representative US population^30^. Another limitation is that our results are correlational results, from which we cannot draw causal relations. However, the findings can be used by policymakers and decision-makers in firms to address the “prediction problem”^3,24^ for people who are ready to receive vaccines. In this respect, decision-makers have to identify characteristics of people in certain regions or firms to target tailored policies. Suppose, they have knowledge about a significantly positive correlation between risk tolerance and the motivation of people that are ready to receive vaccines for reasons of getting access. In this case, decision-makers in firms may use this insight together with data that represents risk attitudes of branches and departments, predicting where they can attract employees by offering vaccination incentives that grant access to certain payment schemes. Since the problem is one of predicting the right target for policies, the policymaker does not need to know what the cause-and-effect relation between risk tolerance and the vaccination motive of getting access is^3,24^. For instance, to increase the motivation of those people, who are in principle ready to become vaccinated, to regularly refresh vaccines, firms may offer “access” to bonus payments conditional that the potential recipient was recently vaccinated. Moreover, the positive correlation between compliance, information about the vaccine and the motive to become vaccinated for protective reasons adds further insights. For firms and federal states, this suggests that information campaigns may help to convince compliant people, who are mostly not vaccine refusers, to become regularly vaccinated. However, care should be taken in the design of the information campaigns, when promoting information on vaccines’ effectiveness and their safety, since this may lead to adverse effects regarding people’s willingness to comply with public-health guidelines^38^. Information campaigns may be meaningful in regions characterized by lower incidence levels. In labor markets, information may help to increase the motivation of becoming regularly vaccinated for those employees, who are not vaccination refusers and who work in non-financial branches (e.g., care facilities), where the employees are typically characterized by a lower appetite for risk. Moreover, the gender effect adds further interesting insights, suggesting that information campaigns may be particularly successful in branches or departments with a high share of female workers - if they are no vaccination refusers. Our findings mark a promising first step to a better understanding of the determinants of vaccination motives of people, who are in principle ready to receive vaccines. We believe that these insights may stimulate further research on the predictive power of economic preferences on individual behavior, which will allow policymakers to target tailored policies in public economics and in labor markets. Improved public policies based on (non-monetary) incentives to increase vaccination rates may work as “gentle rule enforcement”^39^. Gentle rule enforcement may be an interesting alternative to be considered in the recent debate on the introduction of compulsory COVID-19 vaccination in Germany. The start of this debate triggered an emotional discussion, which seems to have the potential to divide the society. A possible path for future research is the elaboration on the motives of vaccine hesitancy and the underlying determinants.

## Supplementary Information

Supplementary Information.

## Data Availability

All data generated or analyzed during this study are included in this published article and its supplementary information files.
