# Supplementary material for: Determinants of people’s motivations to approach COVID-19 vaccination centers
Source: Sci Rep. 2023 Mar 31;13:5282. doi: 10.1038/s41598-023-30244-4 (PMC10064609; doi:10.1038/s41598-023-30244-4)
Supplement: Supplementary file 1 — Supplementary Information. [file 41598_2023_30244_MOESM1_ESM.pdf]

Supplementary Information

Part A - Data

All data are available online at this Git Hub Repository:
https://github.com/hrau1380/vaccination/raw/main/data.xlsx

In accordance with the Declaration of Helsinki, all participants were shown a consent form and agreed with its terms before taking part in our survey. Participants were guaranteed the anonymity of the data generated during the survey. For the GLOBE Lab of the University of Göttingen there is a general IRB approval, which refers to the laboratory. Our study procedure is in line with the ethical guidelines stated in this approval.

Part B - Table

	self-protetction		protection of others		fear of Omicron	
	(1)	(2)	(3)	(4)	(5)	(6)
PC1: Trust others & Political participation	0.171**	0.010	0.141**	0.053	0.129*	0.077
	(0.067)	(0.070)	(0.070)	(0.071)	(0.071)	(0.074)
PC2: Compliant & Feels informed	0.281***	0.269***	0.201***	0.209***	0.096	0.060
	(0.070)	(0.070)	(0.072)	(0.070)	(0.073)	(0.073)
PC3: Altruistic & Impatient	0.003	0.074	0.079	0.190**	-0.016	0.054
	(0.070)	(0.093)	(0.073)	(0.093)	(0.074)	(0.098)
PC4: Risk tolerant	-0.066	-0.125	-0.131*	-0.211***	0.014	-0.057
	(0.070)	(0.078)	(0.073)	(0.077)	(0.074)	(0.081)
female	0.284*	0.205	0.417***	0.351**	0.316**	0.264*
	(0.152)	(0.151)	(0.158)	(0.151)	(0.160)	(0.158)
age		-0.008		-0.178**		0.175**
		(0.084)		(0.084)		(0.088)
student		-0.442		-0.568*		-0.252
		(0.308)		(0.306)		(0.323)
German		0.190		-0.007		0.267
		(0.342)		(0.340)		(0.359)
contact with people at risk		0.036		0.079		0.108
		(0.158)		(0.159)		(0.166)
COVID-19 experience		-0.003		0.074		0.076
		(0.072)		(0.071)		(0.075)
receive booster vaccination		1.325***		1.416***		1.150***
		(0.363)		(0.360)		(0.380)
constant	-0.180	-.0.904	-0.242	-0.943*	-0.196*	-1.570***
	(0.113)	(0.556)	(0.117)	(0.553)	(0.118)	(0.583)
controls for location and wave	no	yes	no	yes	no	yes
obs.	156	154	154	152	156	154
R^2	0.166	0.268	0.152	0.308	0.059	0.166

Standard errors in parentheses
*** p<0.01, ** p<0.05, * p<0.1

Table S1. OLS regressions disaggregated results of the "protection & fear" index.

Part C - Questions of the survey

(Translated from German)

Preferences

[Risk tolerance]

- *How do you assess yourself? Are you generally willing to take risks?* (0 = not at all; 10 = completely)

[Time Preferences]

- *How much money do you want to receive today, such that you give up a sure payment of €1000 in 6 months?* (Please state an amount between €0 and €1000.)
- *How much money do you want to receive in 6 months, such that you give up a sure payment of €1000 in 12 months?* (Please state an amount between €0 and €1000.)

[Altruism]

- *Imagine you are walking down the street. You have exactly ten 1-euro coins in your wallet. You pass a booth of a well-known charity.* (How many of your euro coins (0-10) do you put in the organization's donation box?)

[Trust in others]

- *In general, how much trust do you have in other people?* (0 = not at all; 10 = completely)

[Voting behavior]

- *How important do you think it is to vote?* (0 = totally unimportant; 10 = very important).

Contextual questions on confidence in COVID-19 vaccination

[Information about COVID-19 vaccines]

- *To what extent do you feel correctly informed about the COVID-19 vaccination?* (0 = not at all; 10 = very good)

[Efficacy of the vaccines]

- *How well do you think vaccination protects against COVID-19?* (0 = not at all; 10 = very good)

[Fear of side effects]

- *How worried are you about the side effects of vaccinations?* (0 = not afraid; 10 = very afraid)

[Amount of COVID-19 vaccinations]

- *Is this your booster shot?* (yes/no)

Contextual questions on motivations to receive the COVID-19 vaccination and experiences and behavior in the pandemic

[Motivations to become vaccinated]

Why do you want to be vaccinated today? Please rate the following reasons. (0 = does not apply; 10 = does perfectly apply

- For reasons of self-protection against COVID-19.
- To protect people from COVID-19.
- For reasons of fear of the new Omicron variant from South Africa.
- To access 2G/3G restricted areas.
- To avoid peer or societal pressure.

[Experiences with COVID-19]

- *Have you had a COVID-disease experience?* (0 = not at all ill; 10 = hospital)

[Social distance and compliance]

- *How hard do you try to keep your distance from other people in times of high incidences in public?* (0 = not at all; 10 = completely)

[Contact to people at risk]

- *Do you have regular contact with people who are particularly threatened by COVID?* (yes/no)

Demographics

- *How old are you?*
- *What gender are you?*
- *What is your nationality?*
- *Have you studied or are you studying?*
